# A study of heat and mass transfer in a fractional MHD flow over an infinite oscillating plate

**DOI:** 10.1186/s40064-015-1426-4

**Published:** 2015-10-24

**Authors:** N. Shahid

**Affiliations:** Forman Christian College, A Chartered University, Lahore, Pakistan

**Keywords:** Magnetohydrodynamic viscous fluid, Fractional derivatives, Oscillating flows, Exact solutions, Fox H-functions, General Wright function, Velocity field, Mass concentration, Thermal radiation, Generalized function, Discrete laplace transforms, Error function

## Abstract

Exact expressions of velocity, temperature and mass concentration have been calculated for free convective flow of fractional MHD viscous fluid over an oscillating plate. Expressions of velocity have been obtained both for sine and cosine oscillations of plate. Corresponding fractional differential equations have been solved by using Laplace transform and inverse Laplace transform. The expression of temperature and mass concentration have been presented in the form of Fox-H function and in the form of general Wright function, respectively and velocity is presented in the form of integral solutions using Generalized function. Some limiting cases of fluid and fractional parameters have been discussed to retrieve some solutions present in literature. The influence of thermal radiation, mass diffusion and fractional parameters on fluid flow has been analyzed through graphical illustrations.

## Background

A great deal of efforts have been put to study the phenomena of mass transfer and radiative heat flux in free convective flows because of its applications in many industrial and chemical processes. In principal, many physical and biological configurations and working owe their appearance and existence to mass and heat transfer. In manufacturing and chemical industry, many processes include radiative heat flows such as construction of satellites, oil and other chemicals’ filtration, construction of accessorizes using solar power, nuclear power processes, fuel combustion and drying of porous mediums’, etc. The process of mass transfer is very similar to heat flow in fluids and this similarity has prompted researchers to study both processes simultaneously. In particular, for the cases of low mass transfer and for low concentration in the fluid, the process of mass transfer and heat flow behave in almost the same way. The study of combined effects of mass transfer and radiation of heat in fluid flow has become a central topic of investigation in recent times. Furthermore, the study of motion of magnetohydrodynamic (MHD) fluid bears its own importance in the context of its vast use in many industrial and nuclear processes.

Exact solutions for various Newtonian and non Newtonian fluids over oscillating bodies for various physical settings have been calculated. Penton (1968) seems to be first to have calculated transient solutions for the flow of Newtonian fluid due to an oscillating plate. An interesting problem of fluid flow with effects of radiative heat on fluid motion was presented by Puri and Kythe (1998). A valuable contribution on this line was made by Erdogan (2000) when he obtained the exact solutions for the motion of viscous fluid due to sine and cosine oscillations of a vertical plate. Rajagopal (1983), Rajagopal and Bhatnagar (1995), Hayat et. al. (2001) and Fetecau et. al. (2005, 2006), etc further extended the study of motion of fluids in various geometrical scenarios for sine oscillations, cosine oscillations, longitudinal and torsional oscillations, etc.

Similarly many authors have worked on determining exact solutions for free convection flows. Soundalgekar (1979) was first to have calculated the exact solution of free convective flow over an infinite oscillating plate and this problem was extended by Soundalgekar and Akolkar (1983) by including the effects of mass transfer on fluid motion. Soundalgekar et. al. (1994) and Das et. al. (1994) further studied the effects of mass transfer on flow past an impulsively started and vertical oscillating plate. Thermal radiation effects on a laminar flow was studied by England and Emry (1969) and Gupta and Gupta (1974) presented the exact solutions of motion of electrically conducting fluid with radiation effects in the presence of uniform magnetic field. Mazumdar and Deka (2007) and Muthucumaraswamy (2006) studied the effects of thermal radiation in an MHD flow and on flow past impulsively started plate, respectively.

But the exact solutions of constitutive models of fluid motion with fractional derivatives are rare in present literature. The study of effects of radiation and mass transfer on free convective flows using fractional calculus tools is even more rare and is a motivation for present investigation. Fractional constitutive relationship model has an advantage over customary constitutive relationship model as it readily assesses the properties of viscous and molecular mediums that are sometimes overlooked by ordinary derivative models. Fractional calculus approach is useful when it comes to generalization of complex dynamics of fluid motion. Many authors (Wang and Xu 2009; Fetecau et al. 2010; Tripathi et al. 2010; Hyat et al. 2010a; Hayat et al. 2010b; Liu and Zheng 2011; Fetecau et al. 2011; Jamil et al. 2011a, b; Tripathi et al. 2011a, b; Tripathi 2011a, b; Zheng et al. 2010; Liu et al. 2011; Zheng et al. 2011a, b, 2012) have recently ventured out on studying the motion of viscous MHD fluids using fractional derivatives and made valuable contributions.

The following study is undertaken to investigate the thermal radiation and diffusion effects on a free convective MHD fractional fluid flow over a vertical oscillating plate. Exact expressions of velocity field, temperature and concentration of fluid have been calculated and presented in interesting forms. Also, the expressions of velocity have been obtained both for sine and cosine oscillations of plate. Limiting cases of fractional and fluid parameters have also been taken into account to retrieve new and some existing expressions of velocity, temperature and concentration of fluid. The influence of fluid and fractional parameters on fluid motion have also been depicted through graphs and some similarities and differences of velocity profiles for sine and cosine oscillations have also been highlighted through graphs.

## Mathematical formulation

A case of fractional incompressible viscous MHD fluid over an infinite vertical oscillating plate is considered. We take x-axis of the Cartesian coordinate system along the vertical direction of the infinite plate and y-axis will be normal to the plate. $T_\infty$ and $C_\infty$ are considered to be initial temperature and concentration of plate and fluid, respectively. At time $t=0^+$, the plate is given an oscillating motion in its own plane with the velocity $f_1\cos \omega _1 t$ or $f_1\sin \omega _1 t$. At the same time the temperature and concentration of the plate are raised to $T_W$ and $C_W$, respectively, and magnetic field of uniform strength $B_0$ is applied to the plate in normal direction. It is assumed that magnetic Reynold’s number is very small and the induced magnetic field is negligible in comparison to transverse magnetic field. The viscous dissipation and Soret and Duoffer effects due to lower level of concentration are assumed to be negligible.

Above assumptions and Boussinesq’s approximation lead to the following set of governing equations of unsteady flow

1 $$\begin{aligned} \frac{\partial u(y,t)}{\partial t}=\nu \frac{\partial ^2{u(y,t)}}{\partial y^2}+g\beta (T(y,t)-T_\infty )+g\beta ^*(C(y,t)-C_\infty )-\frac{\sigma B_0^2}{\rho }u(y,t);\,\,\,\,\,y,t>0 \end{aligned}$$

2 $$\begin{aligned} \rho C_P\frac{\partial T(y,t)}{\partial t}=\kappa \frac{\partial ^2{T(y,t)}}{\partial y^2}-\frac{\partial q_r(y,t)}{\partial y};\,\,\,\,\,y,t>0 \end{aligned}$$

3 $$\begin{aligned} \frac{\partial C(y,t)}{\partial t}=D\frac{\partial ^2{C(y,t)}}{\partial y^2};\,\,\,\,\,y,t>0 \end{aligned}$$

and initial and boundary conditions with the assumption of no slip between fluid and plate are

4 $$\begin{aligned} u(y,t)=0,\,\,T(y,t)=T_\infty ,\,\,C(y,t)=C_\infty ,\,\,\,\,\,y\ge 0,\,t=0 \end{aligned}$$

5 $$\begin{aligned} u(0,t)=f_1\cos (\omega _1 t)\,\,or\,\,f_1\sin (\omega _1 t),\,\,T(0,t)=T_W,\,\,C(0,t)= C_\infty +(C_W-C_\infty )\frac{U_0^2 t}{\nu },\,\,t>0 \end{aligned}$$

6 $$\begin{aligned} u(y,t)\rightarrow 0,\,\,T(y,t)\rightarrow T_\infty ,\,\,C(y,t)\rightarrow C_\infty \,\,as\,\,y\rightarrow \infty \end{aligned}$$

where *u*(*y*, *t*),*T*(*y*, *t*), *C*(*y*, *t*),$\nu$, *g*, $\beta$, $\beta ^*$, $\kappa$, $q_r$, $C_P$, $\rho$ and *D* are velocity of the fluid, its temperature, species concentration in the fluid, kinematic viscosity, gravitational acceleration, coefficient of thermal expansion, coefficient of expansion with concentration, thermal conductivity of the fluid, radiative heat flux, specific heat at constant pressure, density of fluid and mass diffusion coefficient, respectively.

Also in Eq. (5), $f_1$ and $U_0$ are constants, and $\omega _1$ is the frequency of oscillation.

Following Cogly-Vincentine-Gilles equilibrium model based on assumption of optically thin medium with relative low density, we have

7 $$\begin{aligned} \frac{\partial q_r(y,t)}{\partial y}=4(T(y,t)-T_\infty )\int _0^\infty K_W \left (\frac{\partial {e_b}}{\partial {T}}\right)_W d\lambda =4I^*(T(y,t)-T_\infty ) \end{aligned}$$

where $K_W$ and $e_b$ are absorption coefficient and plank function.

Introducing Eq. (7) in Eq. (2), we have

8 $$\begin{aligned} \rho C_P\frac{\partial T(y,t)}{\partial t}=\kappa \frac{\partial ^2{T(y,t)}}{\partial y^2}-4I^*(T(y,t)-T_\infty );\,\,\,\,\,y,t>0 \end{aligned}$$

To obtain solutions of Eqs. (1), (3) and (8) along with initial and boundary conditions (4), (5) and (6), we first convert these equations in dimensionless form.

The following dimensionless quantities have been introduced

9 $$\begin{aligned} u^{*}&=\frac{u}{U_0},\,\,\,y^{*}=\frac{yU_0}{\nu },\,\,\,t^{*}=\frac{tU_0^2}{\nu },\,\,\, T^*=\frac{T-T_\infty }{T_W-T_\infty }\nonumber \\ C^*&=\frac{C-C_\infty }{C_W-C_\infty },\,\,\,P_r=\frac{\mu C_P}{\kappa },\,\,\,S_c=\frac{\nu }{D},\,\,\,\,G_r=\frac{\rho \beta \nu (T-T_\infty )}{U_0^3} \nonumber \\ G_m&=\frac{g\beta ^*\nu (C-C_\infty )}{U_0^3},\,\,\,\,M=\frac{\sigma B_0^2\nu }{\rho U_0^2},\,\,\,\,F=\frac{4I^*\nu ^2}{\kappa U_0^2} \end{aligned}$$

where $P_r$, $S_c$, $G_r$, $G_m$, *M* and *F* are Prandtl number, Schmidth number, thermal Grashof number, mass Grashof number, Hartmann number and dimensionless thermal radiation parameter, respectively.

Using dimension less quantities (9) in governing Eqs. (1), (3) and (8) and dropping “*” notation, we obtain

10 $$\begin{aligned} \frac{\partial u(y,t)}{\partial t}=\frac{\partial ^2{u(y,t)}}{\partial y^2}+G_rT(y,t)+G_mC(y,t)-Mu(y,t);\quad y,t>0 \end{aligned}$$

11 $$\begin{aligned} \frac{\partial T(y,t)}{\partial t}=\frac{1}{P_r}\frac{\partial ^2{T(y,t)}}{\partial y^2}-\frac{F}{P_r}T(y,t);\quad  y,t>0 \end{aligned}$$

12 $$\begin{aligned} \frac{\partial C(y,t)}{\partial t}=\frac{1}{S_c}\frac{\partial ^2{C(y,t)}}{\partial y^2}; \quad y,t>0 \end{aligned}$$

The corresponding initial and boundary conditions are

13 $$\begin{aligned} u(y,0)=T(y,0)=C(y,0)=0; \quad y\ge 0 \end{aligned}$$

14 $$\begin{aligned} &u(0,t)=\frac{f_1}{U_0}\cos \omega _1\left(\frac{t^*\nu }{U_0^2}\right)=f\cos (\omega t)\,\,or\,\,f\sin (\omega t),\,\,T(0,t)=1,\,\,C(0,t)=t;\quad t>0&\\\nonumber &u(y,t),T(y,t), C(y,t)\rightarrow 0\,\,\, as\,\,\,y\rightarrow \infty \end{aligned}$$

where $f=\frac{f_1}{U_0}$ is a constant and $\omega =\frac{\omega _1\nu }{U_0^2}$ is new frequency of oscillation.

To obtain analytical formulas for velocity, temperature and concentration, we use fractional derivative approach. In particular, we consider Caputo fractional differential operator. Equations (10), (11) and (12) with Caputo derivative take the form

15 $$\begin{aligned} D_t^\alpha u(y,t)=\frac{\partial ^2{u(y,t)}}{\partial y^2}+G_rT(y,t)+G_mC(y,t)-Mu(y,t); \quad y,t>0 \end{aligned}$$

16 $$\begin{aligned} D_t^\beta T(y,t)=\frac{1}{P_r}\frac{\partial ^2{T(y,t)}}{\partial y^2}-\frac{F}{P_r}T(y,t); \quad y,t>0 \end{aligned}$$

17 $$\begin{aligned} D_t^\gamma C(y,t)=\frac{1}{S_c}\frac{\partial ^2{C(y,t)}}{\partial y^2}, \quad y,t>0 \end{aligned}$$

where Caputo differential operator $D_t^\alpha$ is defined as (Podlubny 1999; Mainardi 2010)

$$\begin{aligned} D_t^\alpha f(t)=\frac{1}{\Gamma (1-\alpha )}\int _o^t{\frac{f'(\tau )}{(t-\tau )^\alpha }d\tau };\,\,\,0<\alpha <1 \end{aligned}$$

where $\Gamma (.)$ is the Gamma function.

## Analytical solutions

Analytical solutions will be obtained by means of Laplace transform and inverse Laplace transform.

Applying Laplace transform to Eq. (17) and using Laplace transform of corresponding initial and boundary condition (13) and (14), we obtain

18 $$\begin{aligned} \bar{C}(y,q)=\frac{1}{q^2}e^{-\sqrt{S_cq^\gamma }y} \end{aligned}$$

where $\bar{C}(y,q)$ is the Laplace transform of *C*(*y*, *t*).

In order to obtain *C*(*y*, *t*), we write Eq. (18) in the form

19 $$\begin{aligned} \bar{C}(y,q)=\frac{1}{q^2}\sum _{n=0}^\infty \frac{(-\sqrt{S_c}y)^n}{n!}q^{\frac{\gamma n}{2}} \end{aligned}$$

Applying Laplace inverse transform to Eq. (19), we obtain

20 $$\begin{aligned} C(y,t)=t\sum _{n=0}^\infty \frac{{\left(\frac{-\sqrt{S_c}y}{t^{\frac{\gamma }{2}}}\right)^n}}{{n!}{\Gamma \left(2-\frac{\gamma n}{2}\right)}} \end{aligned}$$

satisfying initial and boundary conditions for mass concentration of the fluid.

Eq. (20) can also be written in the form of general Wright function i.e.

21 $$\begin{aligned} C(y,t)=tW_{-\frac{\gamma }{2},2}\bigg (\frac{-\sqrt{S_c}y}{t^{\frac{\gamma }{2}}}\bigg ),\,\,\,for\,\,0<\gamma <1 \end{aligned}$$

In above, the general Wright function is defined as (2010)

$$\begin{aligned} W_{\lambda ,\mu }(z)=\sum _{n=0}^\infty \frac{z^n}{n!\Gamma (\lambda n+\mu )},\,\,\,\lambda >-1\,\,,\mu \in \mathcal {C} \end{aligned}$$

Now, applying Laplace transform to Eq. (16) and using Laplace transform of corresponding initial and boundary conditions (13) and (14), we obtain

22 $$\begin{aligned} \bar{T}(y,q)=\frac{1}{q}e^{{-\sqrt{P_rq^\beta +F}y}} \end{aligned}$$

To find $T(y,t)=L^{-1}\{\bar{T}(y,q)\}$, we firstly write Eq. (22) in the following form

23 $$\begin{aligned} \bar{T}(y,q)=\frac{1}{q}+\frac{1}{q}\sum _{n=1}^\infty \frac{(-\sqrt{F}y)^n}{n!}\sum _{j=0}^\infty \frac{\Gamma \left(\frac{n}{2}+1\right)\left(\frac{P_r}{F}\right)^jq^{\beta j}}{j!\Gamma \left(\frac{n}{2}-j+1\right)} \end{aligned}$$

Taking Laplace inverse transform of Eq. (23), we obtain

24 $$\begin{aligned} T(y,t)=1+\sum _{n=1}^\infty \frac{(-\sqrt{F}y)^n}{n!} \sum _{j=0}^\infty \frac{\Gamma \left(\frac{n}{2}+1\right)\left(\frac{P_r}{Ft^\beta }\right)^j}{j!\Gamma \left(\frac{n}{2}-j+1\right)\Gamma \left(1-\beta j\right)} \end{aligned}$$

satisfying initial and boundary conditions of temperature.

We can also write the above expression in terms of Fox-H function,

25 $$\begin{aligned} T(y,t)=1+\sum _{n=1}^\infty \frac{(-\sqrt{F}y)^n}{n!} H^{1,1}_{1,3}\left[ \frac{-P_r}{Ft^\beta }{\bigg|}\begin{array}{l}(-\frac{n}{2},0)\\ (0,1),(-\frac{n}{2},-1),(0,-\beta ) \end{array}\right] \end{aligned}$$

where Fox- H function is defined as (Mathai et al. 2010)

$$\begin{aligned} \mathop \sum _{n=0}^{\infty }\frac{(-z)^n\prod _{j=1}^{p}\Gamma (a_j+A_jn)}{n!\prod _{j=1}^{q}\Gamma (b_j+B_jn)}= H^{1,p}_{p,q+1}\left[ z {\bigg |}\begin{array}{l} (1-a_1,A_1),\dots,(1-a_p,A_p) \\ (0,1),(1-b_1,B_1),\ldots,(1-b_q,B_q) \end{array} \right] . \end{aligned}$$

Now to find the exact expression for velocity field *u*(*y*, *t*), we apply discrete Laplace transform to Eq. (15) and obtain

26 $$\begin{aligned} \frac{\partial ^2{\bar{u}(y,q)}}{\partial y^2}-(q^\alpha +M)\bar{u}(y,q)=-G_r\bar{T}(y,q)-G_m\bar{C}(y,q) \end{aligned}$$

where $\bar{u}(y,q)$ is the Laplace transform of *u*(*y*, *t*). Also, $\bar{u}(y,q)$ has to satisfy the condition

27 $$\begin{aligned} \bar{u}(0,q)=\frac{fq}{q^2+\omega ^2}\,\,or\,\,\frac{f\omega }{q^2+\omega ^2} \end{aligned}$$

Solving Eq. (26) with the help of Eqs. (18), (22) and (27), we obtain

28 $$\begin{aligned} \bar{u}_c(y,q)&=\frac{fqe^{-\sqrt{q^\alpha +M}y}}{q^2+\omega ^2}+\frac{G_r e^{-\sqrt{q^\alpha +M}y}}{q[P_r q^\beta -q^\alpha +(F-M)]} +\frac{G_m e^{-\sqrt{q^\alpha +M}y}}{q^2[S_cq^\gamma -q^\alpha -M]}\nonumber \\ &-\frac{G_re^{-\sqrt{P_rq^\beta +F}y}}{q[P_r q^\beta -q^\alpha +(F-M)]}-\frac{G_me^{-\sqrt{q^\gamma S_c}y}}{q^2[S_cq^\gamma -q^\alpha -M]} \end{aligned}$$

To find $u_c(y,t)=L^{-1}\{\bar{u}_c(y,q)\}$, which is velocity of the fluid corresponding to cosine oscillations of the plate, we apply Laplace inverse transform to Eq. (28) and using Appendix (47), (48), (49) and we obtain analytic expression of velocity field

29 $$\begin{aligned} u_{c}(y,t)&=f\cos (\omega t)+f\sum _{p=0}^\infty (-\omega ^2)^p\sum _{n=1}^\infty \frac{(-y)^n}{n!}t^{2p-\frac{\alpha n}{2}}\nonumber \\&\qquad \sum _{m=0}^\infty \frac{(Mt^{\alpha })^m\Gamma {(\frac{n}{2}+1)}}{m!\Gamma {(\frac{n}{2}+1-m)}\Gamma (1+2p-\frac{\alpha n}{2}+\alpha m)}\\&\quad +\frac{G_r}{P_r}\sum _{p=0}^\infty \left( \frac{1}{P_r}\right) ^p\sum _{n=0}^\infty \frac{(-y)^n}{n!} \sum _{m=0}^\infty \frac{(M)^m\Gamma {(\frac{n}{2}+1)}}{m!\Gamma {(\frac{n}{2}-m+1)}\Gamma (\alpha m-\frac{\alpha n}{2})}\nonumber \\&\qquad \int ^t_0 G_{\beta ,p\alpha ,p+1}\left( \frac{M-F}{P_r},s\right) \left( t-s\right) ^{\alpha m-\frac{\alpha n}{2}}ds \nonumber \\&\quad +\frac{G_m}{S_c}\sum _{p=0}^\infty \left( \frac{1}{S_c}\right) ^p\sum _{n=0}^\infty \frac{(-y)^n}{n!} \sum _{m=0}^\infty \frac{(M)^m\Gamma {(\frac{n}{2}+1)}}{m!\Gamma {(\frac{n}{2}-m+1)}\Gamma (\alpha m-\frac{\alpha n}{2}+1)}\nonumber \\&\qquad \int ^t_0 G_{\gamma ,p\alpha ,p+1}\left( \frac{M}{S_c},s\right) \left( t-s\right) ^{\alpha m-\frac{\alpha n}{2}+1}ds \nonumber \\&\quad -\frac{G_r}{P_r}\sum _{p=0}^\infty \frac{1}{(P_r)^p}\sum _{n=0}^\infty \frac{(-\sqrt{P_r}y)^n}{n!} \sum _{m=0}^\infty \bigg (\frac{F}{P_r}\bigg )^m\frac{\Gamma (\frac{n}{2}+1)}{m!\Gamma (\frac{n}{2}-m+1)\Gamma (\beta m-\frac{\beta n}{2})}\nonumber \\&\qquad \int ^t_0 G_{\beta ,p\alpha -1 ,p+1}\left( \frac{M-F}{P_r}, s\right) \nonumber \\&\quad \times (t-s)^{\beta m-\frac{\beta n}{2}-1}ds-\frac{G_m}{S_c}\sum _{m=0}^\infty \frac{1}{(Sc)^m} \sum _{n=0}^\infty \frac{(-\sqrt{S_c}y)^n}{n!\Gamma (2-\frac{\gamma n}{2})} \nonumber \\&\qquad \int _0^t G_{\gamma ,\alpha m,m+1}\left( \frac{M}{S_c}, s\right) \left( t-s\right) ^{1-\frac{\gamma }{2}n}ds\nonumber \end{aligned}$$

corresponding to cosine oscillations.

Similarly, we obtain an expression of velocity corresponding to sine oscillations of plate i.e.

30 $$\begin{aligned} u_{s}(y,t)&=f\sin (\omega t)+f\omega \sum _{p=0}^\infty (-\omega ^2)^p\sum _{n=1}^\infty \frac{(-y)^n}{n!}t^{2p-\frac{\alpha n}{2}+1}\nonumber \\&\qquad \sum _{m=0}^\infty \frac{(Mt^{\alpha })^m\Gamma {(\frac{n}{2}+1)}}{m!\Gamma {(\frac{n}{2}+1-m)}\Gamma (2+2p-\frac{\alpha n}{2}+\alpha m)} \\&\quad +\frac{G_r}{P_r}\sum _{p=0}^\infty \left( \frac{1}{P_r}\right) ^p\sum _{n=0}^\infty \frac{(-y)^n}{n!} \sum _{m=0}^\infty \frac{(M)^m\Gamma {(\frac{n}{2}+1)}}{m!\Gamma {(\frac{n}{2}-m+1)}\Gamma (\alpha m-\frac{\alpha n}{2})}\nonumber \\&\qquad \int ^t_0 G_{\beta ,p\alpha ,p+1}\left( \frac{M-F}{P_r},s\right) (t-s)^{\alpha m-\frac{\alpha n}{2}}ds \nonumber \\&\quad +\frac{G_m}{S_c}\sum _{p=0}^\infty \left( \frac{1}{S_c}\right) ^p\sum _{n=0}^\infty \frac{(-y)^n}{n!} \sum _{m=0}^\infty \frac{(M)^m\Gamma {(\frac{n}{2}+1)}}{m!\Gamma {(\frac{n}{2}-m+1)}\Gamma (\alpha m-\frac{\alpha n}{2}+1)}\nonumber \\&\qquad \int ^t_0 G_{\gamma ,p\alpha ,p+1}\left( \frac{M}{S_c},s\right) (t-s)^{\alpha m-\frac{\alpha n}{2}+1}ds \nonumber \\&\quad -\frac{G_r}{P_r}\sum _{p=0}^\infty \frac{1}{(P_r)^p}\sum _{n=0}^\infty \frac{(-\sqrt{P_r}y)^n}{n!} \sum _{m=0}^\infty \bigg (\frac{F}{P_r}\bigg )^m\frac{\Gamma (\frac{n}{2}+1)}{m!\Gamma (\frac{n}{2}-m+1)\Gamma (\beta m-\frac{\beta n}{2})}\nonumber \\&\qquad \int ^t_0 G_{\beta ,p\alpha -1 ,p+1}\left( \frac{M-F}{P_r}, s\right) \nonumber \\&\quad \times (t-s)^{\beta m-\frac{\beta n}{2}-1}ds-\frac{G_m}{S_c}\sum _{m=0}^\infty \frac{1}{(Sc)^m} \sum _{n=0}^\infty \frac{(-\sqrt{S_c}y)^n}{n!\Gamma (2-\frac{\gamma n}{2})} \nonumber \\&\qquad \int _0^t G_{\gamma ,\alpha m,m+1}\left( \frac{M}{S_c}, s\right) \left( t-s\right) ^{1-\frac{\gamma }{2}n}ds\nonumber \end{aligned}$$

Velocity expressions (29) and (30) corresponding to cosine oscillations and sine oscillations, respectively can also be written as

31 $$\begin{aligned} u_{c}(y,t)&=f\cos (\omega t)+f\sum _{p=0}^\infty (-\omega ^2)^p\sum _{n=1}^\infty \frac{(-y)^n}{n!}t^{2p-\frac{\alpha n}{2}} H^{1,3}_{1,1}\nonumber \\&\qquad \left[ -Mt^\alpha  { \bigg |} \left( -\frac{n}{2},0\right) {\left( 0,1\right) , \left( -\frac{n}{2},-1\right) , \left( \frac{\alpha n}{2}-2p,\alpha \right) } \right] \\&\quad +\frac{G_r}{P_r}\sum _{p=0}^\infty \left( \frac{1}{P_r}\right) ^p\sum _{n=0}^\infty \frac{(-y)^n}{n!} \sum _{m=0}^\infty \frac{(M)^m\Gamma {(\frac{n}{2}+1)}}{m!\Gamma {(\frac{n}{2}-m+1)}\Gamma (\alpha m-\frac{\alpha n}{2})}\nonumber \\&\qquad \int ^t_0 G_{\beta ,p\alpha ,p+1}\left( \frac{M-F}{P_r},s\right) (t-s)^{\alpha m-\frac{\alpha n}{2}}ds \nonumber \\&\quad +\frac{G_m}{S_c}\sum _{p=0}^\infty \left( \frac{1}{S_c}\right) ^p\sum _{n=0}^\infty \frac{(-y)^n}{n!} \sum _{m=0}^\infty \frac{(M)^m\Gamma {(\frac{n}{2}+1)}}{m!\Gamma {(\frac{n}{2}-m+1)}\Gamma (\alpha m-\frac{\alpha n}{2}+1)}\nonumber \\&\qquad \int ^t_0 G_{\gamma ,p\alpha ,p+1}\left( \frac{M}{S_c},s\right) (t-s)^{\alpha m-\frac{\alpha n}{2}+1}ds \nonumber \\&\quad -\frac{G_r}{P_r}\sum _{p=0}^\infty \frac{1}{(P_r)^p}\sum _{n=0}^\infty \frac{(-\sqrt{P_r}y)^n}{n!} \sum _{m=0}^\infty \bigg (\frac{F}{P_r}\bigg )^m\frac{\Gamma (\frac{n}{2}+1)}{m!\Gamma (\frac{n}{2}-m+1)\Gamma (\beta m-\frac{\beta n}{2})}\nonumber \\&\qquad \int ^t_0 G_{\beta ,p\alpha -1 ,p+1}\left( \frac{M-F}{P_r}, s\right) \nonumber \\&\quad \times (t-s)^{\beta m-\frac{\beta n}{2}-1}ds-\frac{G_m}{S_c}\sum _{m=0}^\infty \frac{1}{(Sc)^m} \sum _{n=0}^\infty \frac{(-\sqrt{S_c}y)^n}{n!\Gamma (2-\frac{\gamma n}{2})}\nonumber \\&\qquad \int _0^t G_{\gamma ,\alpha m,m+1}\left( \frac{M}{S_c}, s\right) (t-s)^{1-\frac{\gamma }{2}n}ds\nonumber \end{aligned}$$

and

32 $$\begin{aligned} u_{s}(y,t)&=f\sin (\omega t)+f\omega \sum _{p=0}^\infty \left( -\omega ^2\right) ^p\sum _{n=1}^\infty \frac{(-y)^n}{n!}t^{2p-\frac{\alpha n}{2}+1}H^{1,3}_{1,1}\nonumber \\&\qquad \left[ -Mt^\alpha {\bigg |} \left( -\frac{n}{2},0\right) {\left( 0,1\right) ,\left( -\frac{n}{2},-1\right) ,\left( \frac{\alpha n}{2}-2p-1,\alpha \right) } \right] \\&\quad +\frac{G_r}{P_r}\sum _{p=0}^\infty \left( \frac{1}{P_r}\right) ^p\sum _{n=0}^\infty \frac{(-y)^n}{n!} \sum _{m=0}^\infty \frac{(M)^m\Gamma {(\frac{n}{2}+1)}}{m!\Gamma {(\frac{n}{2}-m+1)}\Gamma (\alpha m-\frac{\alpha n}{2})}\nonumber \\&\qquad \int ^t_0 G_{\beta ,p\alpha ,p+1}\left( \frac{M-F}{P_r},s\right) (t-s)^{\alpha m-\frac{\alpha n}{2}}ds \nonumber \\&\quad +\frac{G_m}{S_c}\sum _{p=0}^\infty \left( \frac{1}{S_c}\right) ^p\sum _{n=0}^\infty \frac{(-y)^n}{n!} \sum _{m=0}^\infty \frac{(M)^m\Gamma {(\frac{n}{2}+1)}}{m!\Gamma {(\frac{n}{2}-m+1)}\Gamma (\alpha m-\frac{\alpha n}{2}+1)}\nonumber \\&\qquad \int ^t_0 G_{\gamma ,p\alpha ,p+1}\left( \frac{M}{S_c},s\right) (t-s)^{\alpha m-\frac{\alpha n}{2}+1}ds \nonumber \\&\quad -\frac{G_r}{P_r}\sum _{p=0}^\infty \frac{1}{(P_r)^p}\sum _{n=0}^\infty \frac{(-\sqrt{P_r}y)^n}{n!} \sum _{m=0}^\infty \bigg (\frac{F}{P_r}\bigg )^m\frac{\Gamma (\frac{n}{2}+1)}{m!\Gamma (\frac{n}{2}-m+1)\Gamma (\beta m-\frac{\beta n}{2})}\nonumber \\&\qquad \int ^t_0 G_{\beta ,p\alpha -1 ,p+1}\left( \frac{M-F}{P_r}, s\right) \nonumber \\&\quad \times (t-s)^{\beta m-\frac{\beta n}{2}-1}ds-\frac{G_m}{S_c}\sum _{m=0}^\infty \frac{1}{(Sc)^m} \sum _{n=0}^\infty \frac{(-\sqrt{S_c}y)^n}{n!\Gamma (2-\frac{\gamma n}{2})}\nonumber \\&\qquad \int _0^t G_{\gamma ,\alpha m,m+1}\left( \frac{M}{S_c}, s\right) \left( t-s\right) ^{1-\frac{\gamma }{2}n}ds\nonumber \end{aligned}$$

satisfying initial and boundary conditions.

## Limiting cases

For $\alpha ,\,\,\,\beta ,\,\,\,\gamma \rightarrow 1$ in Eqs. (18), (22) and (28), we can retrieve (*y*, *t*) solutions of governing equations in ordinary differential operator. Some significant limiting cases have been discussed below.

### Solution in the absence of magnetic field

The absence of magnetic field i.e. $M=0$ and, assumptions of $\alpha ,\,\,\,\beta ,\,\,\,\gamma \rightarrow 1$ and $f=1$ in Eq. (28) lead to the following expression corresponding to cosine oscillations

33 $$\begin{aligned} \bar{u}_c(y,q)=\frac{qe^{-\sqrt{q}y}}{q^2+\omega ^2}+\frac{G_r e^{-\sqrt{q}y}}{q[P_r q-q+F]} +\frac{G_m e^{-\sqrt{q}y}}{q^2[S_cq-q]} -\frac{G_re^{-\sqrt{P_r q+F}y}}{q[P_r q-q+F]}-\frac{G_me^{-\sqrt{q S_c}y}}{q^2[S_cq-q]} \end{aligned}$$

Applying Laplace inverse transform to Eq. (33) and using Appendix (50) and (51), we obtain velocity for cosine oscillations i.e.

34 $$\begin{aligned} u_c(y,t)&=\int ^t_0\frac{ye^{-\frac{y^2}{4s}}}{2\sqrt{\pi }s^{\frac{3}{2}}}\cos {\omega (t-s)}ds+\frac{G_r}{(P_r-1)}\int ^t_{0}erfc\left( \frac{y}{2\sqrt{s}}\right) e^{-\frac{F}{P_r-1}{(t-s)}}ds \nonumber \\&\quad +\frac{G_m}{(S_c-1)}\int ^t_{0}erfc\left( \frac{y}{2\sqrt{s}}\right) (t-s)ds+\frac{G_r\sqrt{P_r}y}{2F\sqrt{\pi }}\int ^t_{0}\frac{e^{-\frac{P_r y^2}{4s}-\frac{F}{P_r}s}}{s^{\frac{3}{2}}}ds \nonumber \\&\quad -\frac{G_r \sqrt{P_r}y}{2F\sqrt{\pi }}\int ^t_{0}\frac{e^{-\frac{P_r y^2}{4s}-\frac{F}{P_r-1}(t-s)-\frac{F}{P_r}s}}{s^{\frac{3}{2}}}ds -\frac{G_m}{(S_c-1)}\int ^t_{0}erfc\left( \frac{\sqrt{S_c}y}{2\sqrt{s}}\right) (t-s)ds \end{aligned}$$

Eq. (34) can be further simplified using Appendix (52) and (53) and the following expressions

$$\begin{aligned} \int ^t_0\frac{ye^{-\frac{y^2}{4s}}}{2\sqrt{\pi }s^{\frac{3}{2}}}\cos {\omega (t-s)}ds=\frac{1}{2}Re e^{i\omega t} \bigg [e^{\sqrt{i\omega }y}erfc\left( \frac{y}{2\sqrt{t}}+{\sqrt{i\omega }y}\right) +e^{-\sqrt{i\omega }y}erfc(\frac{y}{2\sqrt{t}}-{\sqrt{i\omega }y})\bigg ] \end{aligned}$$

$$\begin{aligned} \frac{\sqrt{P_r}y}{2\sqrt{\pi }}\int ^t_{0}\frac{e^{-\frac{P_r y^2}{4s}-\frac{F}{P_r}s}}{s^{\frac{3}{2}}}ds= \frac{1}{2} \bigg [e^{\sqrt{F}y}erfc\bigg (\frac{\sqrt{P_r}y}{2\sqrt{t}}-\sqrt{\frac{Ft}{P_r}}\bigg )+ e^{-\sqrt{F}y}erfc\bigg (\frac{{\sqrt{P_r}y}}{2\sqrt{t}}+\sqrt{\frac{Ft}{P_r}}\bigg )\bigg ] \end{aligned}$$

$$\begin{aligned} \frac{\sqrt{P_r}y}{2\sqrt{\pi }}\int ^t_{0}\frac{e^{-\frac{P_r y^2}{4s}-\frac{F}{P_r-1}(t-s)-\frac{F}{P_r}s}}{s^{\frac{3}{2}}}ds= \frac{1}{2} \bigg [e^{-i\sqrt{\frac{F}{P_r-1}}y}erfc\bigg (\frac{{\sqrt{P_r}y}}{2\sqrt{t}}-i\sqrt{\frac{Ft}{P_r(P_r-1)}}\bigg ) \\ \nonumber +e^{i\sqrt{\frac{F}{P_r-1}}y}erfc\bigg (\frac{{\sqrt{P_r}y}}{2\sqrt{t}}+i\sqrt{\frac{Ft}{P_r(P_r-1)}}\bigg )\bigg ] \end{aligned}$$

$$\begin{aligned} \int ^t_{0}erfc(\frac{y}{2\sqrt{s}})e^{-\frac{F}{P_r-1}{(t-s)}}ds=\frac{P_r-1}{F}\bigg \{erfc(\frac{y}{2\sqrt{t}})- \frac{e^{-\frac{F}{P_r-1}t}}{2} \bigg [e^{-i\sqrt{\frac{F}{P_r-1}}y}erfc\bigg (\frac{y}{2\sqrt{t}}-i\sqrt{\frac{Ft}{P_r-1}}\bigg ) \nonumber \\ +e^{i\sqrt{\frac{F}{P_r-1}}y}erfc\bigg (\frac{y}{2\sqrt{t}}+i\sqrt{\frac{Ft}{P_r-1}}\bigg )\bigg ]\bigg \} \end{aligned}$$

Similarly, velocity corresponding to sine oscillation is

35 $$\begin{aligned} u_s(y,t)&=\int ^t_0\frac{ye^{-\frac{y^2}{4s}}}{2\sqrt{\pi }s^{\frac{3}{2}}}\sin {\omega (t-s)}ds+\frac{G_r}{(P_r-1)}\int ^t_{0}erfc\left( \frac{y}{2\sqrt{s}}\right) e^{-\frac{F}{P_r-1}(t-s)}ds \nonumber \\&\quad +\frac{G_m}{(S_c-1)}\int ^t_{0}erfc\left( \frac{y}{2\sqrt{s}}\right) (t-s)ds+\frac{G_r\sqrt{P_r y}}{2F\sqrt{\pi }}\int ^t_{0}\frac{e^{-\frac{P_r y^2}{4s}-\frac{F}{P_r}s}}{s^{\frac{3}{2}}}ds \nonumber \\&\quad -\frac{G_r\sqrt{P_r} y}{2F\sqrt{\pi }}\int ^t_{0}\frac{e^{-\frac{P_r y^2}{4s}-\frac{F}{P_r-1}(t-s)-\frac{F}{P_r}s}}{s^{\frac{3}{2}}}ds -\frac{G_m}{(S_c-1)}\int ^t_{0}erfc\left( \frac{\sqrt{S_c}y}{2\sqrt{s}}\right) (t-s)ds \end{aligned}$$

where

$$\begin{aligned} \int ^t_0\frac{ye^{-\frac{y^2}{4s}}}{2\sqrt{\pi }s^{\frac{3}{2}}}\sin {\omega (t-s)}ds=\frac{1}{2}Img e^{i\omega t} \bigg [e^{\sqrt{i\omega }y}erfc(\frac{y}{2\sqrt{t}}+{\sqrt{i\omega }y})+e^{-\sqrt{i\omega }y}erfc\left( \frac{y}{2\sqrt{t}}-{\sqrt{i\omega }y}\right) \bigg ] \end{aligned}$$

We observe that both velocity expressions $u_c(y,t)$ and $u_s(y,t)$ satisfy initial and boundary conditions even in the absence of magnetic field.

### Solution in the case of constant radiative heat flux and $\beta \rightarrow 1$

Assuming radiative heat flux to be constant along y-direction of plate, F = 0 (or $q_r$ = constant)and $\beta \rightarrow 1$, we obtain from Eq. (22) the following expression

36 $$\begin{aligned} \bar{T}(y,q)=\frac{1}{q}e^{{-\sqrt{P_rq}y}} \end{aligned}$$

Applying Laplace inverse transform to Eq. (36) and using Appendix (51), we obtain an expression for temperature of the fluid in the absence of thermal radiation i.e.

37 $$\begin{aligned} T(y,t)=erfc\left( \frac{\sqrt{P_r}y}{2\sqrt{t}}\right) \end{aligned}$$

satisfying also the corresponding boundary condition (14) for temperature where *erfc*(.) represents complementary error function.

### Solution in the absence of magnetic field and constant radiative heat flux

The absence of magnetic field, $M=0$, constant radiative heat flux along y-direction of plate, F = 0 (or $q_r$ = constant) and assumption of $\alpha ,\,\,\,\beta ,\,\,\,\gamma \rightarrow 1$ in Eq. (33) lead to the following expression

38 $$\begin{aligned} \bar{u}_c(y,q)=\frac{qe^{-\sqrt{q}y}}{q^2+\omega ^2}+\frac{G_r e^{-\sqrt{q}y}}{q[P_r q-q]} +\frac{G_m e^{-\sqrt{q}y}}{q^2[S_cq-q]} -\frac{G_re^{-\sqrt{P_r q}y}}{q[P_r q-q]}-\frac{G_me^{-\sqrt{q S_c}y}}{q^2[S_cq-q]} \end{aligned}$$

Applying Laplace inverse transform to above expression and using Appendix (51), we obtain velocity for cosine oscillations i.e.

39 $$\begin{aligned} u_c(y,t) &=\int ^t_0\frac{ye^{-\frac{y^2}{4s}}}{2\sqrt{\pi }s^{\frac{3}{2}}}\cos {\omega (t-s)}ds +\frac{G_r}{(P_r-1)}\int ^t_{0}erfc\left( \frac{y}{2\sqrt{s}}\right) ds \nonumber \\ &\quad+\frac{G_m}{(S_c-1)}\int ^t_{0}erfc(\frac{y}{2\sqrt{s}})(t-s)ds \nonumber \\&\quad -\frac{G_r}{(P_r-1)}\int ^t_{0}erfc\left( \frac{\sqrt{P_r}y}{2\sqrt{s}}\right) ds \\ &\quad-\frac{G_m}{(S_c-1)}\int ^t_{0}erfc\left( \frac{\sqrt{S_c}y}{2\sqrt{s}}\right) (t-s)ds \end{aligned}$$

Similarly, velocity corresponding to sine oscillation is

40 $$\begin{aligned} u_s(y,t)&=\int ^t_0\frac{ye^{-\frac{y^2}{4s}}}{2\sqrt{\pi }s^{\frac{3}{2}}}\sin {\omega (t-s)}ds\\ &\quad+\frac{G_r}{(P_r-1)}\int ^t_{0}erfc\left( \frac{y}{2\sqrt{s}}\right) ds +\frac{G_m}{(S_c-1)}\int ^t_{0}erfc\left( \frac{y}{2\sqrt{s}}\right) (t-s)ds \nonumber \\&\quad -\frac{G_r}{(P_r-1)}\int ^t_{0}erfc\left( \frac{\sqrt{P_r}y}{2\sqrt{s}}\right) ds -\frac{G_m}{(S_c-1)}\int ^t_{0}erfc\left( \frac{\sqrt{S_c}y}{2\sqrt{s}}\right) (t-s)ds \end{aligned}$$

where the value of *f* is assumed to be 1.

### Mass concentration corresponding to $\mathbf {\gamma \rightarrow 1}$

Assuming $\gamma \rightarrow 1$ in Eq. (18), we obtain

41 $$\begin{aligned} \bar{C}(y,q)=\frac{1}{q^2}e^{-\sqrt{S_cq}y} \end{aligned}$$

Applying Laplace inverse transform to above expression, we obtain mass concentration for ordinary MHD free convective fluid i.e.

42 $$\begin{aligned} C(y,t)=\int _0^t \frac{y e^{-\frac{-y^2}{4s}}}{2\sqrt{\pi }s^{\frac{3}{2}}}(t-s)ds \end{aligned}$$

which can be further simplified by using Appendix (52) i.e.

43 $$\begin{aligned} C(y,t)=\bigg (t+\frac{y^2 S_c}{2}\bigg )erfc\left( \frac{\sqrt{S_c}y}{2\sqrt{s}}\right) - \frac{y\sqrt{S_c t}}{\sqrt{\pi }}e^{-\frac{-y^2 S_c}{4t}} \end{aligned}$$

or from Eqs. (20) and (21), mass concentration can simply be written as

44 $$\begin{aligned} C(y,t)=t\sum _{n=0}^\infty \frac{{\bigg (\frac{-\sqrt{S_c}y}{\sqrt{t}}\bigg )^n}}{{n!}{\Gamma (2-\frac{ n}{2})}}= tW_{-\frac{1}{2},2}\bigg (\frac{-\sqrt{S_c}y}{\sqrt{t}}\bigg ) \end{aligned}$$

## Results and discussion

Many interesting physical aspects of radiative heat flow have been brought into light through graphs. These graphs also represent the influence of physical parameters $G_r$, $G_m$, $S_c$, $P_r$, *M*, *F*, $\omega$ and fractional parameters $\alpha$, $\beta$, $\gamma$ on motion of MHD fluid over a vertical oscillating plate.

**Fig. 1:**
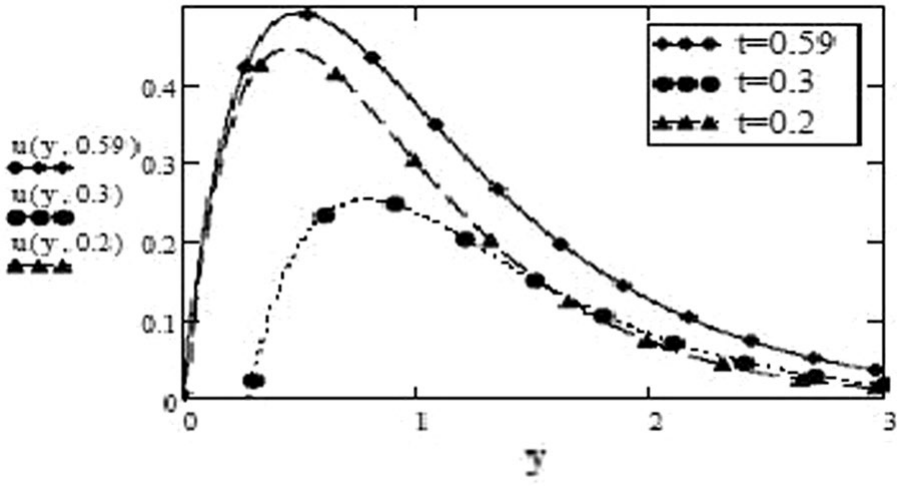
Velocity profiles for different values of t at $G_r = 10$, $G_m = 5$, $S_c = 2.5$, $M = 0.5$, $F = 2.5$, $\omega = 8$, $P_r = 7$, $\alpha = 0.5$, $\beta = 0.3$, $\gamma = 0.2$, $f = 1$ for cosine oscillation

**Fig. 2:**
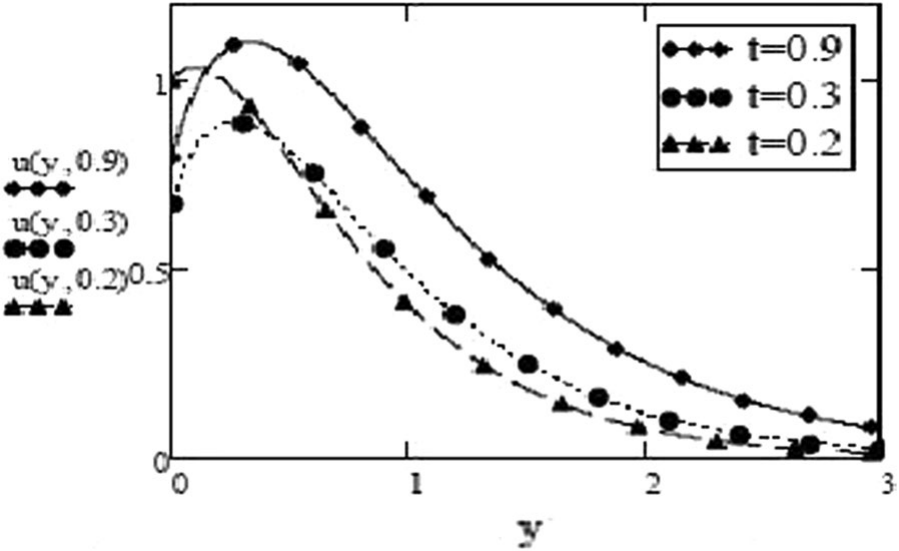
Velocity profiles for different values of t at $G_r = 10$, $G_m = 5$, $S_c = 2.5$, $M = 0.5$, $F = 2.5$, $P_r = 7$, $\omega = 8$, $\alpha = 0.5$, $\beta = 0.3$, $\gamma = 0.2$, $f = 1$ for sine oscillation

Figures 1 and 2 represent velocity profiles for different values of *t* ad for fixed values of $G_r$, $G_m$, $S_c$, $P_r$, *M*, *F*, $\alpha$, $\beta$, $\gamma$, *f* and $\omega$ for sine and cosine oscillations. It can be observed that near the plate for starting time, the velocity profiles override each other but the velocity increases eventually for increasing values of time *t*. Also, it can be seen in both graphs that velocity is vanishing for higher values of *y* as was expected because the impact of oscillations on fluid gets weaker as fluid gets farther away from the plate.

**Fig. 3:**
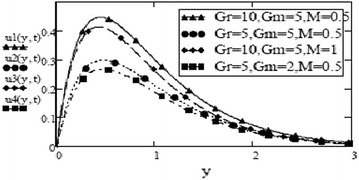
Velocity profiles for different values of $G_r, G_m$ and *M* at t = 0.2, $S_c = 2.5$, $F = 2.5$, $P_r = 7$, $\omega = 8$, $\alpha = 0.5$, $\beta = 0.3$, $\gamma = 0.2$, $f = 1$ for cosine oscillation

**Fig. 4:**
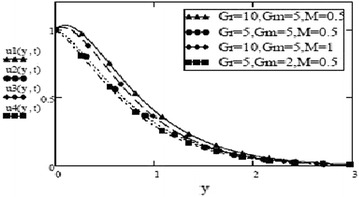
Velocity profiles for different values of $G_r, G_m$ and *M* at t = 0.2, $S_c = 2.5$, $F = 2.5$, $\omega = 8$, $P_r = 7$, $\alpha = 0.5$, $\beta = 0.3$, $\gamma = 0.2$, $f = 1$ for sine oscillation

**Fig. 5:**
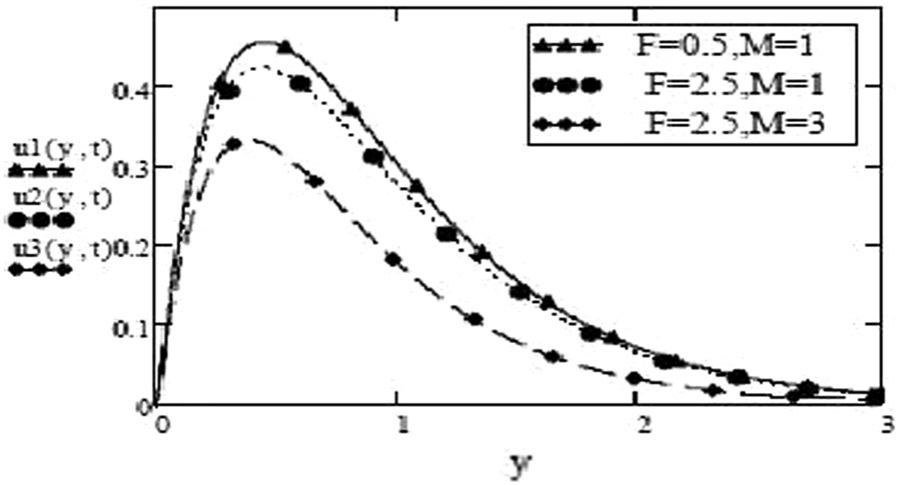
Velocity profiles for different values of F and M at = 0.2 $G_r =10, G_m = 5$, $S_c = 1.5$, $P_r = 7$, $\omega = 8$, $\alpha = 0.5$, $\beta = 0.3$, $\gamma = 0.2$, $f = 1$ for cosine oscillation

**Fig. 6:**
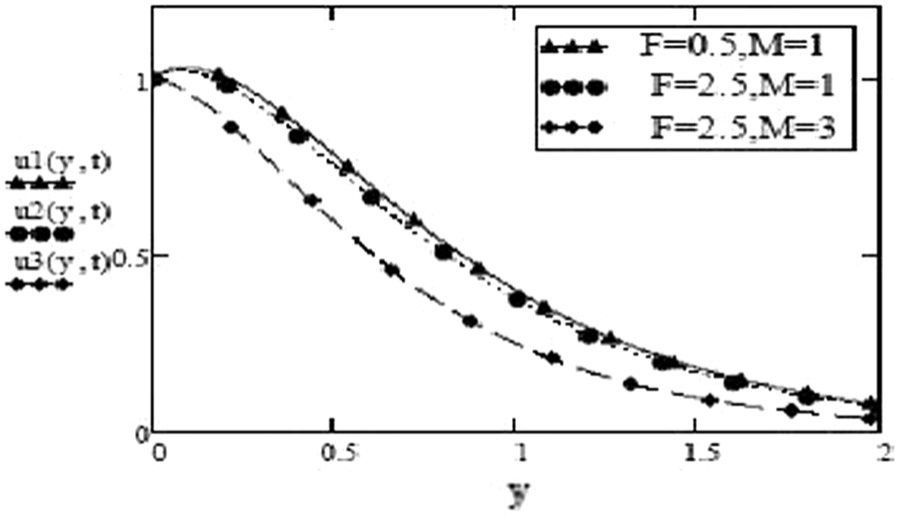
Velocity profiles for different values of F and M at = 0.2 $G_r =10, G_m = 5$, $S_c = 1.5$, $P_r = 7$, $\omega = 8$, $\alpha = 0.5$, $\beta = 0.3$, $\gamma = 0.2$, $f = 1$ for sine oscillation

**Fig. 7:**
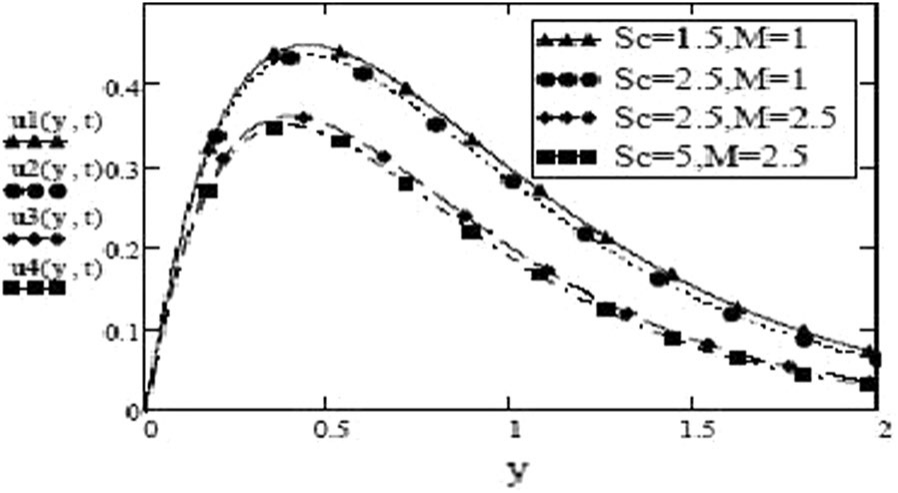
Velocity profiles for different values of $S_c$ and *M* at t = 0.2 $G_r =10, G_m = 5$, $P_r = 7$, $\omega = 8$, $\alpha = 0.5$, $\beta = 0.3$, $\gamma = 0.2$, $f = 1$ for cosine oscillation

**Fig. 8:**
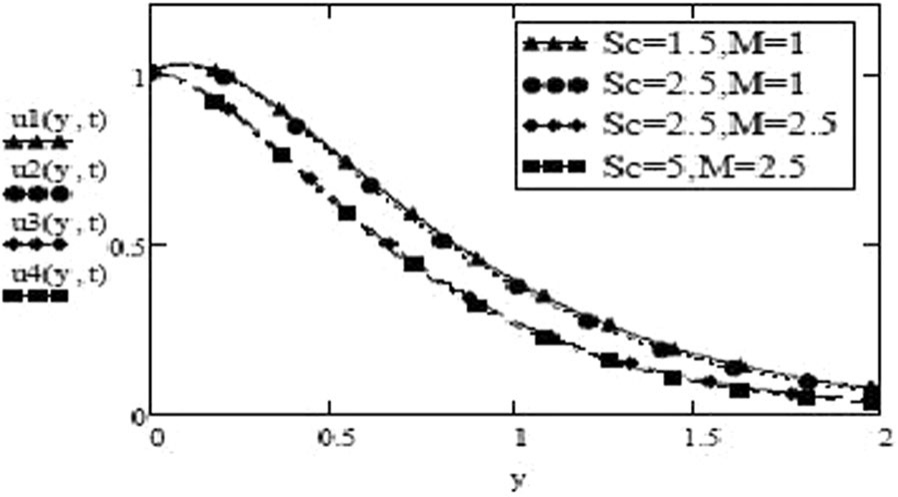
Velocity profiles for different values of $S_c$ and *M* at t = 0.2 $G_r =10, G_m = 5$,$F = 1$, $P_r = 7$, $\omega = 8$, $\alpha = 0.5$, $\beta = 0.3$, $\gamma = 0.2$, $f = 1$ for sine oscillation

Figures 3 and 4 make comparison between velocity profiles for varying values of thermal Grashof number, $G_r$, mass Grashof number, $G_m$ and Hartmann number, *M* and other fluid and fractional parameters are taken to be fixed. Besides the different shape of velocity profiles for sine oscillations and cosine oscillations, it is observed that velocity increases with increase in $G_r$ and $G_m$ and decreases with increase in *M*. The influence of parameters *M*, *F* and $S_c$ on free convective fluid motion has been depicted through Figs. 5, 6, 7 and 8. All these graphs point to the fact that velocity has inverse relation with Schmidth number, $S_c$, Harmann number, *M* and thermal radiation parameter, F even if the pattern of velocity profiles is different for sine and cosine oscillations. It is also noted that velocity responds to the changes of *M* faster than the changes in $S_c$ and *F*.

**Fig. 9:**
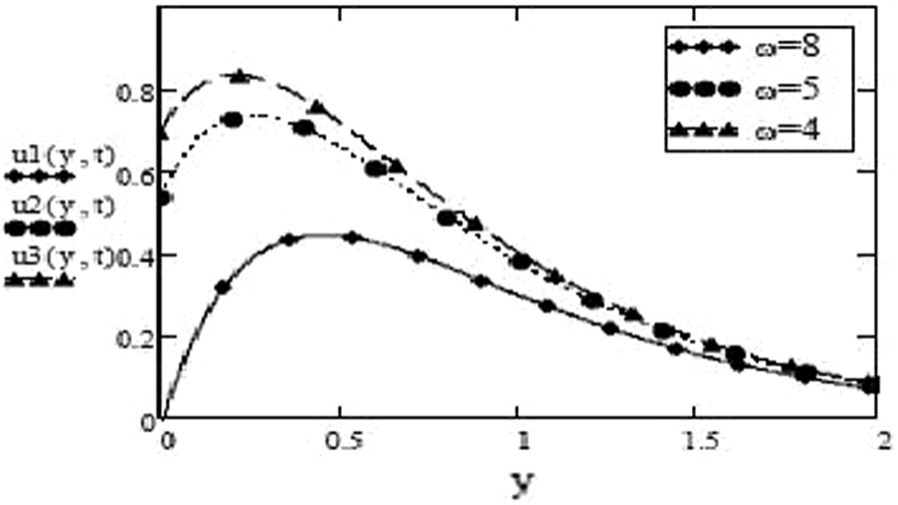
Velocity profiles for different values of oscillating frequency at = 02 $G_r =10, G_m = 5$,$S_c = 2.5$, $P_r = 7$,$F = 2.5$, $M = 0.5$, $\alpha = 0.5$, $\beta = 0.3$, $\gamma = 0.2$, $f = 1$ for cosine oscillation

**Fig. 10:**
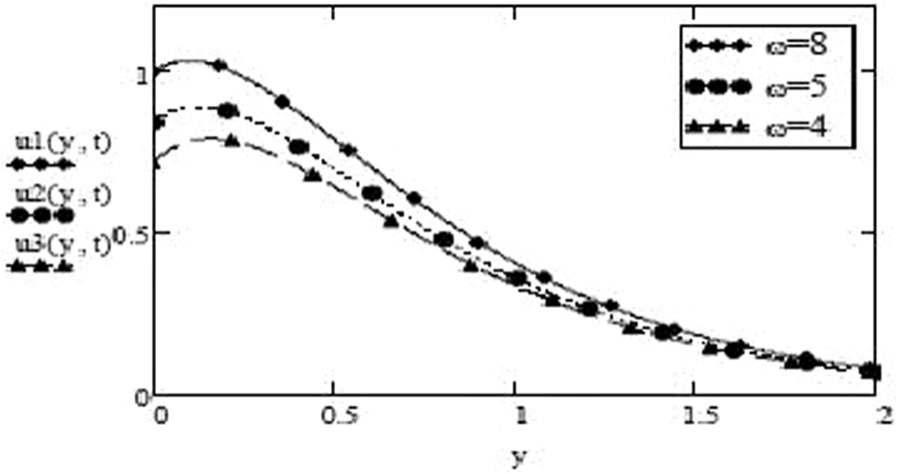
Velocity profiles for different values of oscillating frequency at = 02 $G_r =10, G_m = 5$,$S_c = 2.5$, $P_r = 7$,$F = 2.5$, $M = 0.5$, $\alpha = 0.5$, $\beta = 0.3$, $\gamma = 0.2$, $f = 1$ for sine oscillation

**Fig. 11:**
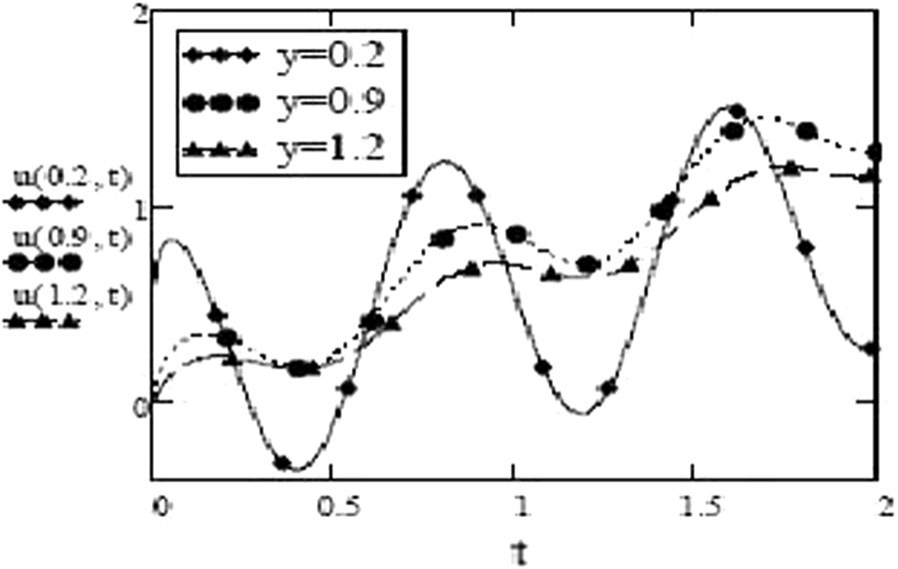
Velocity profiles for different values of *y* at $G_r =10, G_m = 5$,$S_c = 2.5$, $P_r = 7$,$F = 2.5$, $M = 0.5$, $\alpha = 0.5$, $\beta = 0.3$, $\gamma = 0.2$, $\omega = 8$, $f = 1$ for cosine oscillation

**Fig. 12:**
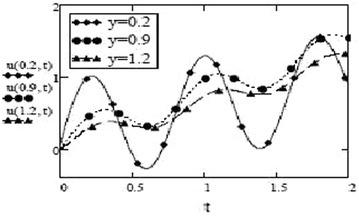
Velocity profiles for different values of *y* at $G_r =10, G_m = 5$,$S_c = 2.5$, $P_r = 7$,$F = 2.5$, $M = 0.5$, $\alpha = 0.5$, $\beta = 0.3$, $\gamma = 0.2$, $\omega = 8$, $f = 1$ for sine oscillation

**Fig. 13:**
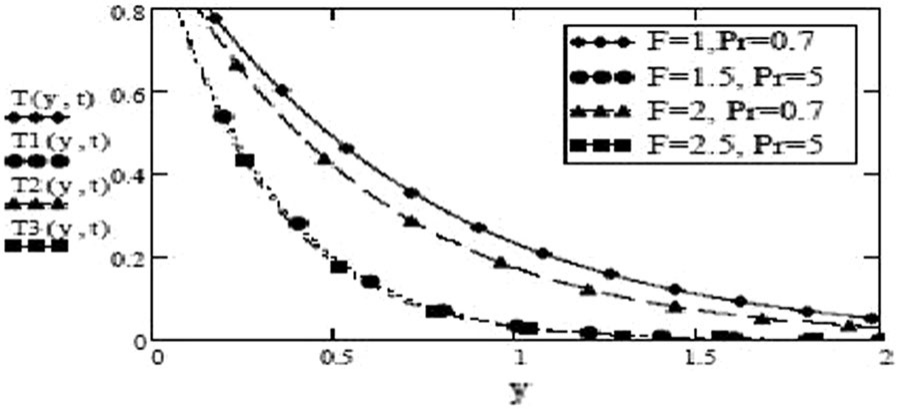
Temperature profiles for different values of F and $P_r$ M at = 0.2 $\beta = 0.5$

Figures 9 and 10 show a contrasting behavior of velocity profiles for cosine oscillations and for sine oscillations. It is observed that velocity is increasing for decreasing values of oscillating frequency of plate for the case of cosine oscillations and in the case of sine oscillations, it is decreasing with decrease in oscillating frequency. However, it is apparent that in both cases, velocity profiles don’t show much different behavior for bigger values of oscillating frequency. Figures 11 and 12 verify the fact that amplitude of oscillations of velocity field decrease with gradual increase in height. Also, it is observed from Fig. 13 that temperature is influenced negatively by Prandtl number, $P_r$ and thermal radiation parameter, *F* i.e. increasing values of $P_r$ and *F* decrease the temperature of fluid.

**Fig. 14:**
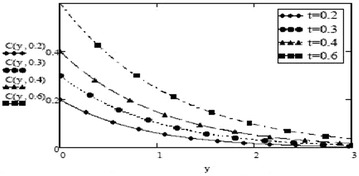
Concentration profiles for different values of t at $S_c = 0.5$ and $\gamma = 0.5$

**Fig. 15:**
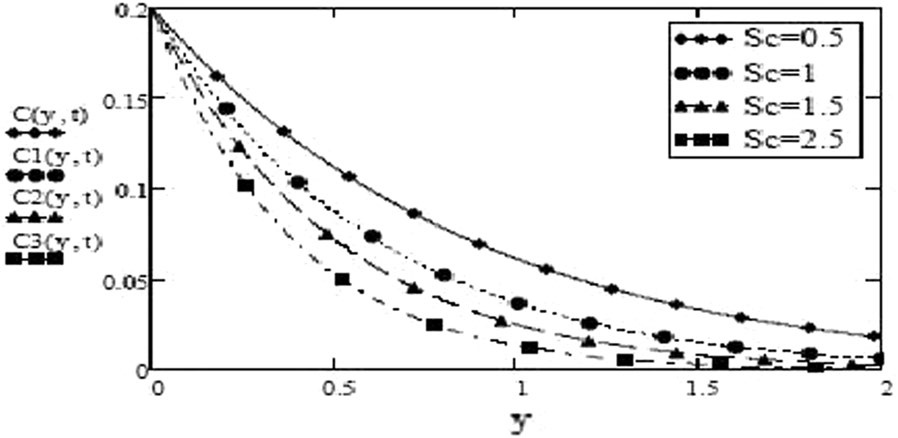
Concentration profiles for different values of $S_c$ at t = 0.2 and $\gamma = 0.5$

**Fig. 16:**
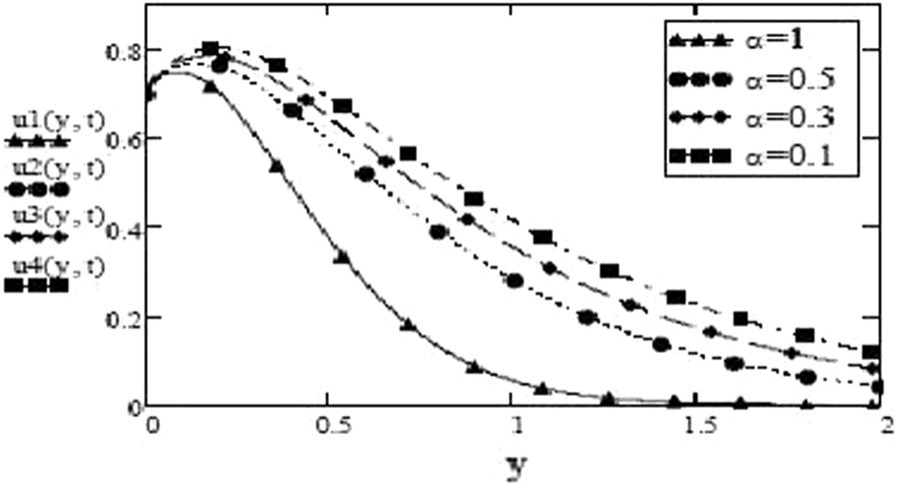
Velocity profiles for different values of $\alpha$ at = 0.1 $G_r =10, G_m = 5$, $M = 0.5$, $S_c = 2.5$, $F = 2.5$, $P_r = 7$, $\beta = 0.3$, $\gamma = 0.2$, $\omega = 8$, $f = 1$ for cosine oscillation

**Fig. 17:**
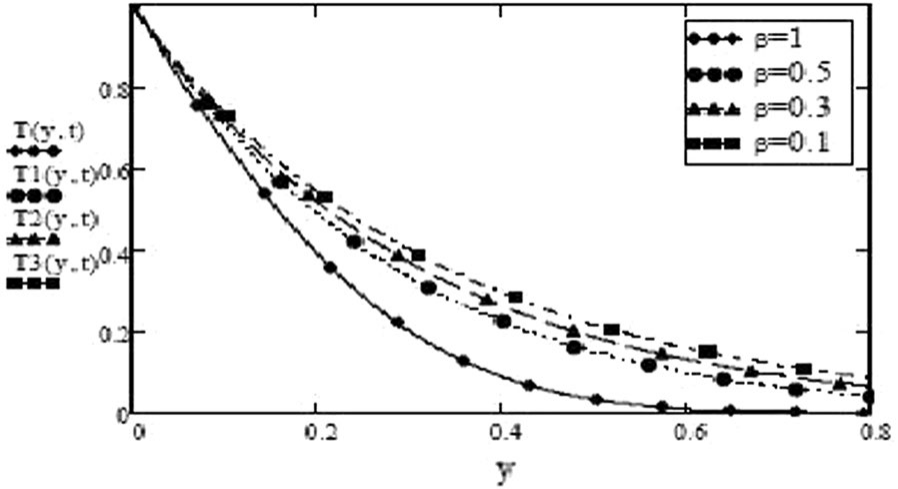
Temperature profiles for different values of $\beta$ at =0.2 $P_r = 7$ F=1.5

**Fig. 18:**
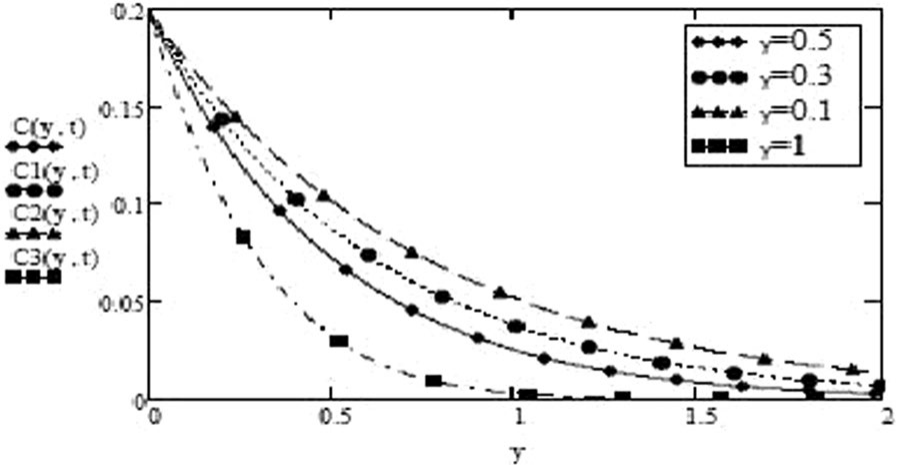
Concentration profiles for different values of $\gamma$ at t = 0.2 $S_c = 0.5$

It can be seen from Figs. 14 and 15 that concentration of fluid increases with increasing time but increasing values of Schmidth number, $S_c$ have negative impact on concentration of fluid. Figures 1, 2, 3, 4, 5, 6, 7, 8, 9, 10, 11 and 12 validate the boundary condition $u(y,t)\rightarrow 0$ as $y\rightarrow \infty$. Influence of fractional parameters on fluid motion is studied through Figs. 16, 17 and 18. These graphs clearly show that velocity, temperature and concentration of fluid decrease for increasing values of fractional parameters $\alpha$, $\beta$ and $\gamma$, respectively. In Figs. 17 and 18, we have also retrieved profiles of temperature and concentration for ordinary MHD convective flow over an oscillating plate by assuming $\beta \rightarrow 1$ and $\gamma \rightarrow 1$.

Finally, in order to have a clearer idea about the accuracy of analytical solutions that have been established, a comparison between the numerical and analytical results was prepared for concentration. The corresponding results have been included in Table 1. The concentration values resulting from Eq. (20), where $n=55$ terms of the sum have been taken into consideration, are compared with those obtained using the Stehfest,s numerical algorithm (Stehfest 1970) for calculating the inverse Laplace transform of the function given by Eq. (18). This algorithm is based on the next relation

45 $$\begin{aligned} C(y,t)=L^{-1}\{\bar{C}(y,q)\}\approx \frac{\ln 2}{t}\sum _{j=1}^{2p}d_j\bar{C}\bigg (y,j\frac{\ln 2}{t}\bigg ), \end{aligned}$$

where *p* is a positive integer,

46 $$\begin{aligned} d_j=(-1)^{j+p}\sum _{k=\bigg [\frac{j+1}{2}\bigg ]}^{min(j,p)}\frac{k^p(2k)!}{(p-k)!k!(k-1)!(j-k)!(2k-j)!} \end{aligned}$$

and [*r*] denotes the integer part of the real number r. According to Table 1, the absolute error being of order $10^{-6}$, there exists a good agreement of the numerical results. Similar comparisons can be made for temperature and velocity.

**Table 1:** Values of concentration C(y, t) resulting from the analytic solution Eq. (20) and the numerical algorithm applied to Eq. (18) at t = 5, Sc = 1 and $\gamma = 0.591$

y	C(y,t), Eq. (20)	C(y,t) Eq. (18)	Absolute error
0	5	5.00001	$6.031\times 10^{-6}$
0.1	4.669	4.669	$3.15\times 10^{-6}$
0.2	4.35855	4.35856	$1.008\times 10^{-5}$
0.3	4.06747	4.06747	$8.068\times 10^{-6}$
0.4	3.79465	3.79465	$6.578\times 10^{-6}$
0.5	3.53903	3.53903	$7.199\times 10^{-6}$
0.6	3.29961	3.29961	$4.718\times 10^{-6}$
0.7	3.07544	3.07544	$4.313\times 10^{-7}$
0.8	2.86562	2.86562	$6.646\times 10^{-6}$
0.9	2.66929	2.6693	$7.367\times 10^{-6}$
1	2.48566	2.48566	$2.036\times 10^{-6}$

## Conclusion

Exact solutions have been calculated for fractional MHD free convective viscous fluid over a vertical oscillating plate and influence of thermal radiation and mass diffusion on fluid motion have been analyzed. Expressions of velocity field, temperature and mass concentration of fluid have been obtained by applying Laplace transform to fractional differential equations governing present fluid flow problem. In particular, Caputo fractional differential operator is favored and motivation to employ fractional calculus tool is generalization of dynamics of such fluid flow problems.

Expressions of velocity field have been obtained for both sine and cosine oscillations of plate and are presented in series form and in the form of integral solutions. The part of velocity corresponding to oscillations of plate is nicely presented in the form of Fox- H function and the part of velocity corresponding to thermal radiation, mass diffusion and magnetic field effects has been presented in integral solutions form, employing the concept of Generalized function. The expression of mass concentration of fractional MHD fluid has been presented in the form of general Wright function and the exact expression of temperature is written in the form of Fox- H function form.

All solutions satisfy initial and boundary conditions.

Some significant limiting cases of fractional and fluid parameters have also been taken into account and expressions of mass concentration and temperature, present in literature, have been retrieved for $\gamma \rightarrow 1$ and, $\beta \rightarrow 1$ and $F=0$, respectively. Also, velocity field expression has been separately calculated for the case when magnetic field is absent as well as for the case of absence of thermal radiation.

To analyze the behavior and influence of fluid and fractional parameters on free convective flow, graphs of velocity, temperature and mass concentration have been drawn and following observations are made:

1. The velocity of fluid for both sine and cosine oscillations increase with increasing *t*, eventually.
2. The velocity has inverse relation with fluid parameters Hartmann number, *M*, thermal radiation parameter, *F* and Schmidth number, $S_c$ and has direct relation with thermal Grashof number, $G_r$ and mass Grashof number, $G_m$.
3. Temperature of fluid increases for decreasing values of Prandtl number, $P_r$ and thermal radiation parameter, *F*.
4. Mass concentration of fluid is negatively influenced by Schmidth number, $S_c$ but it increases with increasing time.
5. A contrasting behavior of velocity profiles for different values of oscillating frequency, $\omega$ for both cases of sine and cosine oscillations has been noted through graphs. These graphs show that the velocity is decreasing for increasing values of oscillating frequency for cosine oscillations and decreases for decreasing frequency for sine oscillations.
6. The influence of fractional parameters on fluid motion is also studied through graphs. These graphs show that for decreasing values of $\alpha$, $\beta$ and $\gamma$, velocity, temperature and concentration increase, respectively.
7. For concentration of fluid *C*(*y*, *t*), the accuracy of obtained analytical solutions has been checked by making a comparison between the numerical and analytical results. Numerical data is in good agreement with analytical results. Same can be done for temperature and velocity field.

## Appendix

47 $$\begin{aligned} L^{-1}\left\{\frac{q^b}{(q^a-d)^c}\right\}=G_{a,b,c}(d,t);\,\,\,\,Re(ac-b)>0,\,\,Re(q)>0,\,\,|\frac{p}{q^a}|<1, \end{aligned}$$

48 $$\begin{aligned} \frac{1}{P_r q^\beta -q^\alpha +(F-M)}=\frac{1}{P_r}\sum _{p=0}^\infty \frac{\left( \frac{1}{P_r}\right) ^p q^{p\alpha }}{\left( q^\beta -\frac{M-F}{P_r}\right) ^{p+1}}, \end{aligned}$$

49 $$\begin{aligned} \frac{1}{S_cq^\gamma -q^\alpha -M}=\frac{1}{S_c}\sum _{m=0}^\infty \frac{\left( \frac{1}{S_c}\right) ^m q^{m\alpha }}{\left( q^\gamma -\frac{M}{S_c}\right) ^{m+1}}, \end{aligned}$$

50 $$\begin{aligned} L^{-1}\{{e^{-\sqrt{q}y}}\}=\frac{ye^{-\frac{y^2}{4t}}}{2\sqrt{\pi }t^{\frac{3}{2}}}, \end{aligned}$$

51 $$\begin{aligned} L^{-1}\bigg \{\frac{e^{-\sqrt{P_rq}y}}{q}\bigg \}=erfc\bigg (\frac{\sqrt{p_r}y}{2\sqrt{t}}\bigg ), \end{aligned}$$

52 $$\begin{aligned} \frac{2y}{\sqrt{\pi }}\int ^t_{0}\frac{e^{b^2 s-\frac{y^2}{s}}}{s^{\frac{3}{2}}}ds= \bigg [e^{-2iby}erfc\bigg (\frac{y}{\sqrt{t}}-ib\sqrt{t}\bigg )+ e^{2iby}erfc\bigg (\frac{y}{\sqrt{t}}+ib\sqrt{t}\bigg )\bigg ], \end{aligned}$$

53 $$\begin{aligned} erf(f(\alpha ))=\frac{2}{\sqrt{\pi }}\int _{f(\alpha )}^\infty e^{-x^2}dx \end{aligned}$$
